# Supplementary material for: Multi‐dataset identification of innovative feature genes and molecular mechanisms in keratoconus
Source: J Cell Mol Med. 2024 Sep 19;28(18):e70079. doi: 10.1111/jcmm.70079 (PMC11412914; doi:10.1111/jcmm.70079)
Supplement: Supplementary file 2 — Material S2. [file JCMM-28-e70079-s002.zip › 7.GSEA analysis/3.DBNDD1/KEGG_SPLICEOSOME.html]

Details for gene set KEGG\_SPLICEOSOME[GSEA]

|  || Dataset | DBNDD1.group.cls#h\_versus\_l |
| Phenotype | group.cls#h\_versus\_l |
| Upregulated in class | h |
| GeneSet | KEGG\_SPLICEOSOME |
| Enrichment Score (ES) | 0.21314195 |
| Normalized Enrichment Score (NES) | 0.900388 |
| Nominal p-value | 0.537037 |
| FDR q-value | 1.0 |
| FWER p-Value | 1.0 |
Table: GSEA Results Summary

  

Fig 1: Enrichment plot: KEGG\_SPLICEOSOME      
 Profile of the Running ES Score & Positions of GeneSet Members on the Rank Ordered List

  

| SYMBOL | TITLE | RANK IN GENE LIST | RANK METRIC SCORE | RUNNING ES | CORE ENRICHMENT || 1 | HSPA2 | na | 5905 | 0.153 | -0.1307 | Yes |
| 2 | PHF5A | na | 7755 | 0.109 | -0.1558 | Yes |
| 3 | HSPA8 | na | 7790 | 0.108 | -0.1286 | Yes |
| 4 | HSPA1L | na | 7968 | 0.104 | -0.1065 | Yes |
| 5 | THOC1 | na | 8312 | 0.098 | -0.0908 | Yes |
| 6 | ACIN1 | na | 8433 | 0.096 | -0.0692 | Yes |
| 7 | PRPF40A | na | 9162 | 0.083 | -0.0686 | Yes |
| 8 | CDC40 | na | 10448 | 0.065 | -0.0887 | Yes |
| 9 | SRSF8 | na | 10874 | 0.060 | -0.0854 | Yes |
| 10 | RBM25 | na | 11100 | 0.057 | -0.0770 | Yes |
| 11 | SRSF6 | na | 11141 | 0.057 | -0.0634 | Yes |
| 12 | LSM4 | na | 11878 | 0.048 | -0.0720 | Yes |
| 13 | PRPF19 | na | 12704 | 0.041 | -0.0852 | Yes |
| 14 | HSPA1A | na | 13025 | 0.038 | -0.0845 | Yes |
| 15 | SRSF3 | na | 13113 | 0.037 | -0.0773 | Yes |
| 16 | ISY1 | na | 13114 | 0.037 | -0.0676 | Yes |
| 17 | SF3B3 | na | 13278 | 0.036 | -0.0629 | Yes |
| 18 | U2AF1 | na | 13390 | 0.035 | -0.0570 | Yes |
| 19 | THOC2 | na | 14000 | 0.030 | -0.0667 | Yes |
| 20 | SF3B5 | na | 14462 | 0.027 | -0.0731 | Yes |
| 21 | HNRNPA1L2 | na | 14677 | 0.025 | -0.0727 | Yes |
| 22 | LSM3 | na | 14740 | 0.025 | -0.0681 | Yes |
| 23 | NCBP1 | na | 15287 | 0.021 | -0.0784 | Yes |
| 24 | ZMAT2 | na | 15355 | 0.020 | -0.0751 | Yes |
| 25 | RBM17 | na | 15424 | 0.020 | -0.0719 | Yes |
| 26 | PPIE | na | 15528 | 0.019 | -0.0699 | Yes |
| 27 | PLRG1 | na | 15543 | 0.019 | -0.0654 | Yes |
| 28 | TRA2A | na | 15831 | 0.017 | -0.0692 | Yes |
| 29 | PRPF6 | na | 15881 | 0.017 | -0.0663 | Yes |
| 30 | U2SURP | na | 15939 | 0.016 | -0.0637 | Yes |
| 31 | PRPF40B | na | 16209 | 0.014 | -0.0677 | Yes |
| 32 | NCBP2 | na | 16211 | 0.014 | -0.0639 | Yes |
| 33 | SNRPD1 | na | 16511 | 0.013 | -0.0693 | Yes |
| 34 | SRSF1 | na | 16662 | 0.012 | -0.0706 | Yes |
| 35 | DDX46 | na | 16817 | 0.011 | -0.0723 | Yes |
| 36 | MAGOH | na | 16827 | 0.011 | -0.0698 | Yes |
| 37 | LSM7 | na | 16856 | 0.010 | -0.0679 | Yes |
| 38 | TXNL4A | na | 16955 | 0.010 | -0.0682 | Yes |
| 39 | CWC15 | na | 17125 | 0.009 | -0.0709 | Yes |
| 40 | SNRPD2 | na | 17207 | 0.008 | -0.0711 | Yes |
| 41 | LSM2 | na | 17720 | 0.005 | -0.0846 | Yes |
| 42 | SLU7 | na | 17728 | 0.005 | -0.0836 | Yes |
| 43 | SF3A1 | na | 17731 | 0.005 | -0.0824 | Yes |
| 44 | ALYREF | na | 17739 | 0.005 | -0.0814 | Yes |
| 45 | SNRPE | na | 17749 | 0.005 | -0.0804 | Yes |
| 46 | SRSF2 | na | 17774 | 0.004 | -0.0800 | Yes |
| 47 | SNRPG | na | 17792 | 0.004 | -0.0794 | Yes |
| 48 | PRPF18 | na | 17883 | 0.004 | -0.0810 | Yes |
| 49 | PPIH | na | 17968 | 0.003 | -0.0827 | Yes |
| 50 | EFTUD2 | na | 18181 | 0.002 | -0.0884 | Yes |
| 51 | HSPA1B | na | 18247 | 0.001 | -0.0900 | Yes |
| 52 | BUD31 | na | 18374 | 0.000 | -0.0936 | Yes |
| 53 | PRPF8 | na | 18475 | -0.001 | -0.0963 | Yes |
| 54 | THOC3 | na | 18488 | -0.001 | -0.0965 | Yes |
| 55 | CRNKL1 | na | 18547 | -0.001 | -0.0979 | Yes |
| 56 | CCDC12 | na | 18572 | -0.001 | -0.0982 | Yes |
| 57 | SNRNP40 | na | 18636 | -0.002 | -0.0995 | Yes |
| 58 | LSM5 | na | 18770 | -0.003 | -0.1027 | Yes |
| 59 | RBM8A | na | 18994 | -0.004 | -0.1079 | Yes |
| 60 | DHX8 | na | 19130 | -0.005 | -0.1104 | Yes |
| 61 | SRSF5 | na | 19132 | -0.005 | -0.1090 | Yes |
| 62 | SNRPB2 | na | 19159 | -0.006 | -0.1083 | Yes |
| 63 | MAGOHB | na | 19177 | -0.006 | -0.1072 | Yes |
| 64 | SNRPF | na | 19199 | -0.006 | -0.1062 | Yes |
| 65 | HNRNPU | na | 19213 | -0.006 | -0.1050 | Yes |
| 66 | HNRNPA1 | na | 19285 | -0.007 | -0.1053 | Yes |
| 67 | LSM8 | na | 19349 | -0.007 | -0.1051 | Yes |
| 68 | XAB2 | na | 19639 | -0.010 | -0.1109 | Yes |
| 69 | SRSF10 | na | 19821 | -0.011 | -0.1132 | Yes |
| 70 | RBM22 | na | 19835 | -0.012 | -0.1106 | Yes |
| 71 | DDX23 | na | 19960 | -0.013 | -0.1108 | Yes |
| 72 | PRPF4 | na | 19973 | -0.013 | -0.1078 | Yes |
| 73 | SART1 | na | 20125 | -0.014 | -0.1085 | Yes |
| 74 | PRPF31 | na | 20138 | -0.014 | -0.1052 | Yes |
| 75 | SF3B6 | na | 20191 | -0.015 | -0.1028 | Yes |
| 76 | SF3B1 | na | 20257 | -0.015 | -0.1007 | Yes |
| 77 | HNRNPA3 | na | 20292 | -0.015 | -0.0977 | Yes |
| 78 | U2AF2 | na | 20442 | -0.017 | -0.0976 | Yes |
| 79 | CDC5L | na | 20655 | -0.019 | -0.0988 | Yes |
| 80 | AQR | na | 20780 | -0.020 | -0.0972 | Yes |
| 81 | CTNNBL1 | na | 20809 | -0.020 | -0.0927 | Yes |
| 82 | SNRPC | na | 20823 | -0.020 | -0.0877 | Yes |
| 83 | HNRNPM | na | 20849 | -0.021 | -0.0830 | Yes |
| 84 | DDX42 | na | 20920 | -0.021 | -0.0795 | Yes |
| 85 | SF3A2 | na | 20930 | -0.021 | -0.0741 | Yes |
| 86 | DHX38 | na | 20988 | -0.022 | -0.0700 | Yes |
| 87 | PUF60 | na | 20990 | -0.022 | -0.0643 | Yes |
| 88 | SNW1 | na | 21042 | -0.022 | -0.0599 | Yes |
| 89 | SNRNP70 | na | 21139 | -0.023 | -0.0566 | Yes |
| 90 | SNRPA | na | 21152 | -0.023 | -0.0508 | Yes |
| 91 | DDX39B | na | 21199 | -0.024 | -0.0459 | Yes |
| 92 | SRSF9 | na | 21205 | -0.024 | -0.0399 | Yes |
| 93 | SNRPB | na | 21223 | -0.024 | -0.0341 | Yes |
| 94 | LSM6 | na | 21259 | -0.024 | -0.0287 | Yes |
| 95 | HNRNPK | na | 21570 | -0.028 | -0.0304 | Yes |
| 96 | DDX5 | na | 21785 | -0.030 | -0.0288 | Yes |
| 97 | PPIL1 | na | 21870 | -0.031 | -0.0232 | Yes |
| 98 | TRA2B | na | 21874 | -0.031 | -0.0152 | Yes |
| 99 | SF3B2 | na | 21892 | -0.031 | -0.0076 | Yes |
| 100 | PQBP1 | na | 21945 | -0.032 | -0.0008 | Yes |
| 101 | SNRPD3 | na | 22046 | -0.033 | 0.0048 | Yes |
| 102 | USP39 | na | 22049 | -0.033 | 0.0132 | Yes |
| 103 | HNRNPC | na | 22107 | -0.033 | 0.0202 | Yes |
| 104 | BCAS2 | na | 22351 | -0.036 | 0.0225 | Yes |
| 105 | DHX16 | na | 22666 | -0.039 | 0.0237 | Yes |
| 106 | SNRNP200 | na | 22754 | -0.040 | 0.0317 | Yes |
| 107 | WBP11 | na | 22797 | -0.041 | 0.0411 | Yes |
| 108 | PRPF38A | na | 22833 | -0.041 | 0.0509 | Yes |
| 109 | PRPF38B | na | 23194 | -0.046 | 0.0524 | Yes |
| 110 | SNRPA1 | na | 23358 | -0.048 | 0.0601 | Yes |
| 111 | DHX15 | na | 23393 | -0.048 | 0.0717 | Yes |
| 112 | PCBP1 | na | 23466 | -0.049 | 0.0824 | Yes |
| 113 | SF3A3 | na | 23702 | -0.052 | 0.0892 | Yes |
| 114 | EIF4A3 | na | 23781 | -0.053 | 0.1009 | Yes |
| 115 | SNRNP27 | na | 23938 | -0.055 | 0.1108 | Yes |
| 116 | SRSF4 | na | 24112 | -0.058 | 0.1209 | Yes |
| 117 | SRSF7 | na | 24128 | -0.058 | 0.1357 | Yes |
| 118 | SMNDC1 | na | 24561 | -0.064 | 0.1400 | Yes |
| 119 | SYF2 | na | 24807 | -0.068 | 0.1506 | Yes |
| 120 | RBMX | na | 24973 | -0.070 | 0.1642 | Yes |
| 121 | CHERP | na | 25082 | -0.072 | 0.1798 | Yes |
| 122 | TCERG1 | na | 25173 | -0.073 | 0.1964 | Yes |
| 123 | PRPF3 | na | 25270 | -0.075 | 0.2131 | Yes |
| 124 | HSPA6 | na | 26391 | -0.095 | 0.2057 | No |
| 125 | SF3B4 | na | 28165 | -0.135 | 0.1897 | No |
Table: GSEA details [plain text format]

  

Fig 2: KEGG\_SPLICEOSOME      
 Blue-Pink O' Gram in the Space of the Analyzed GeneSet

  

Fig 3: KEGG\_SPLICEOSOME: Random ES distribution      
 Gene set null distribution of ES for **KEGG\_SPLICEOSOME**
